# The similarity of inherited diseases (II): clinical and biological similarity between the phenotypic series

**DOI:** 10.1186/s12920-020-00793-y

**Published:** 2020-09-24

**Authors:** Alessio Gamba, Mario Salmona, Laura Cantù, Gianfranco Bazzoni

**Affiliations:** 1 Department of Biochemistry and Molecular Pharmacology, Istituto di Ricerche Farmacologiche Mario Negri IRCCS, Via Mario Negri 2, I-20156 Milan, Italy; 2 Department of Medical Biotechnology and Translational Medicine, University of Milan, LITA, Segrate, Italy

**Keywords:** Gene mutations, Inherited diseases, Disease phenotypes, Biological processes, Network analysis, Ontologies

## Abstract

**Background:**

Despite being caused by mutations in different genes, diseases in the same phenotypic series are clinically similar, as reported in Part I of this study. Here, in Part II, we hypothesized that the phenotypic series too might be clinically similar. Furthermore, on the assumption that gene mutations indirectly cause clinical phenotypes by directly affecting biological functions, we hypothesized that clinically similar phenotypic series might be biologically similar as well.

**Methods:**

To test these hypotheses, we generated a clinical similarity network and a set of biological similarity networks. In both types of network, the nodes represent the phenotypic series, and the edges linking the nodes indicate the similarity of the linked phenotypic series. The weight of each edge is proportional to a similarity coefficient, which depends on the clinical phenotypes and the biological features that are shared by the linked phenotypic series, in the clinical and biological similarity networks, respectively.

**Results:**

After assembling and analyzing the networks, we raised the threshold for the similarity coefficient, to retain edges of progressively greater weight. This way all the networks were gradually split into fragments, composed of phenotypic series with increasingly greater degrees of similarity. Finally, by comparing the fragments from the two types of network, we defined subsets of phenotypic series with varying types and degrees of clinical and biological correlation.

**Conclusions:**

Like the individual diseases, the phenotypic series too are clinically and biologically similar to each other. Furthermore, our findings unveil different modalities of correlation between the clinical manifestations and the biological features of the inherited diseases.

## Background

A ‘multi-level’ study of disease (D), i.e., from the clinical level (of systems and organs) to the biological level (of cells and molecules), offers a reasonable chance of better understanding the underlying mechanisms of D. This type of study has become more feasible than in the past thanks to the availability of comprehensive and detailed databases. For instance, Human Phenotype Ontology (HPO) enlists the disease phenotypes (DP), i.e., the symptoms and signs of the D [1], while Disease Ontology (DO) classifies the D in clinical and anatomical categories [2]. Online Mendelian Inheritance in Man (OMIM) provides information on the D and the mutated genes [3], while Gene Ontology (GO) annotates the biological characteristics of the corresponding gene products [4].

While the multi-level study of individual D is undoubtedly interesting, a comprehensive study of all the molecularly-defined D should facilitate the identification of recurrent biological mechanisms that may become dysfunctional in molecularly dissimilar (albeit clinically similar) D. An attractive hypothesis is that mutations in different disease gene products (DGP) interfere with the same biological mechanism and therefore result in clinically similar DP. This is supported by the frequent clinical similarity even among D that are caused by mutations in biochemically unrelated genes [5]. For this analysis, we employed a very useful feature of the OMIM database [3], the ‘Phenotypic Series’ (PS); these are subsets of clinically similar D (see Additional file 1 for an explanation of the terms). In the first part of the study,^1^ we analyzed the similarity of D belonging to the same PS (*intra*-PS similarity). Then here, in the second part, we analyze the similarity among the PS themselves (*inter*-PS similarity).

Among the tools available for this analysis, we used networks, because they serve to study D at different levels of complexity, thus conceptually linking the clinical manifestations of the D with its molecular determinants [6–8]. In general, networks, which are composed of nodes linked by edges, give a comprehensive display of complex systems, which comprise numerous elements and their interactions [9]. From a network perspective, a PS can be thought of as a meta-node representing a group of similar D. This assumption greatly facilitates the analysis of the interactions among all the elements involved (D, DP, biological characteristics, gene products and genes). We generated two types of network, the Clinical Similarity Network (CSN) and various Biological Similarity Networks (BSN). In both types, each node stands for a PS and each edge (linking a pair of nodes) represents the degree of PS-PS similarity, as defined by the HPO-based clinical DP (in the CSN) and the GO-based biological features (in the BSNs), which the two PS in the pair share.

**Fig. 1:**
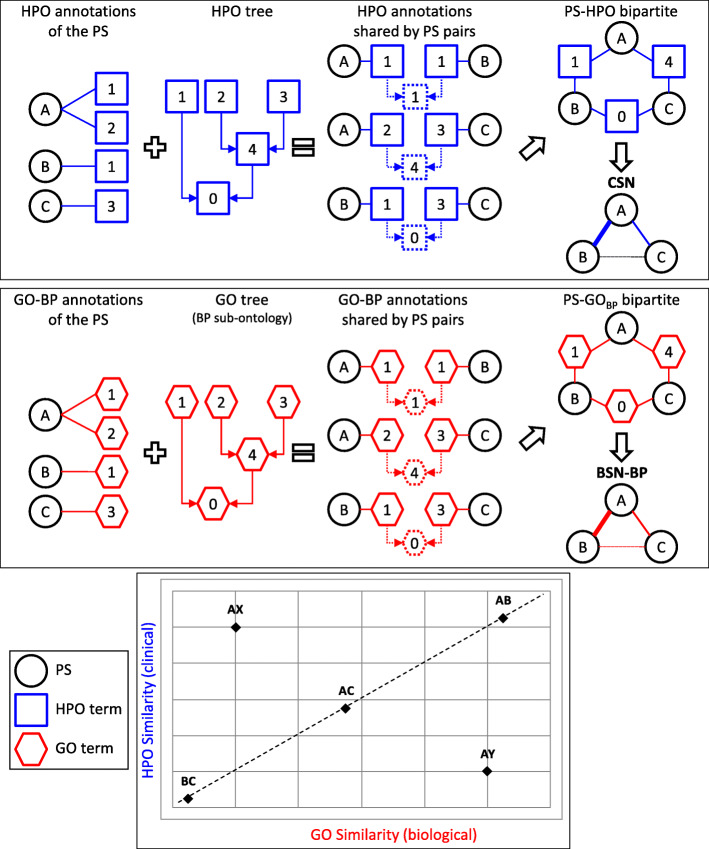
Assembly of the CSN and BSN. The experimental procedure leading to the assembly of the CSN and one of the three BSN (here the BSN-BP) is depicted schematically, in the *top* and *middle* panels, respectively. The PS (*black circles*), labeled A, B and C, are annotated with the HPO terms (*blue squares*) and the GO-BP terms (*red hexagons*) labeled 0, 1, 2, 3 and 4. As shown in the schematic ontology trees, 4 is the common ancestor of terms 2 and 3, and 0 is the root term. Then the algorithm retrieves all the possible PS-PS pairs and the annotations that the two PS share (*dotted squares* and *hexagons*). The shared annotations can be either an identical term (e.g., A and B sharing 1) or the most informative common ancestor of two different terms (e.g., A and C sharing 4 as a common ancestor of 2 and 3); highly dissimilar PS (e.g., B and C) share the root 0. For simplicity’s sake, each pair of PS is assumed here to share only one annotation. At the end of the search, a non-weighted bipartite graph is assembled. Then, from the PS-HPO and PS-GO bipartite graphs, the CSN and the various BSN are derived, by linking all the PS that share the annotations. The *thickness* of the edge in the CSN and BSN is proportional to the similarity coefficients of the linked PS pair. The *bottom* panel shows schematically different types of correlation between HPO- and GO-based similarities. For instance, in the AB, AC and BC pairs of PS, both the clinical and the biological similarities are directly correlated in a high, intermediate or low manner, respectively. In contrast, two additional PS pairs exemplify instances of high clinical (but low biological) similarity (AX) and, conversely, high biological (but low clinical) similarity (AY)

The starting point for assembling all these networks is a bipartite graph, i.e., a network containing two subsets of nodes. We first assembled a PS-HPO bipartite graph (Fig. 1*top panel*), by first linking any PS to nodes representing the clinical DP (as HPO terms) that annotate the D within that PS. Then, in a pairwise comparison of all the PS, we retrieved the HPO annotations that the two PS in each pair share (either as identical term or as the most informative common ancestor of two different terms). Finally, from the bipartite PS-HPO graph, we derived the CSN, using an edge to link all the PS that share HPO annotations (for details, see Additional file 2).

Following the same strategy, we assembled three PS-GO bipartite graphs (Fig. 1*middle panel*), in which each PS is linked to nodes representing functional annotations (as GO terms), as defined in one of the three sub-ontologies of GO, i.e., Biological Process (BP), Cellular Component (CC) and Molecular Function (MF). Then, from the PS-GO_BP_, PS-GO_CC_ and PS-GO_MF_ bipartite graphs, we derived the BSN-BP, BSN-CC and BSN-MF networks, respectively, by linking all the PS that share annotations in the related sub-ontology of GO with an edge. Finally, to display the strongest evidence from the three sub-ontology networks together, we assembled the general BSN, which assigns to each edge only the greatest weight among the three networks.

All the similarity networks are very dense structures, with numerous edges linking each node to all the others. However, the edges have different strengths that correspond to different degrees of similarity. The concept of edge strength reflects the hierarchical structure of the ontology trees. In particular, in both HPO and GO (like in any other ontology), the terms are organized hierarchically as descendants of less specific ancestor terms, down to the least specific common ancestor, the ‘root’ of the tree, which contains all the terms in the ontology. Thus, even two highly dissimilar PS will share at least one term – the root – and possibly some other ancestors of low specificity as well. Consequently, each PS is connected (in the CSN or in the various BSN) to all the other PS, even though many links only represent shared ancestors of rather low specificity. Thus, to focus only on the strongest links, we assigned a weight to each edge, proportional to a calculated similarity coefficient (a measure of how similar two PS are). Then, to remove the weaker links, we progressively raised the thresholds for the coefficients of (clinical or biological) similarity. This way, we defined groups of PS with increasingly greater degrees of (clinical or biological) similarity. Finally, after examining the clinical and biological similarity independently of each other, we directly compared the two types of similarity for each PS pair (Fig. 1*bottom panel*). This enabled us to define different modes of correlation (or lack thereof) between the clinical and biological levels of similarity of the D.

## Methods

### Data and databases

We retrieved the data from the following databases. (i) OMIM [3] for the PS, the PS-associated D and the D-associated DGP; (ii) HPO [1] for the clinical DP of the D; (iii) GO and its three sub-ontologies [10] for the biological features of the DGP; (iv) DO [2] for the clinical categories of the D. In addition, from HPO, GO and DO we retrieved not only the term annotations but also the ontologies, i.e., the hierarchical relations that link each term to its parent and ancestor terms, as well as to its child and descendant terms.

### Generation of a network

To generate the CSN, we proceeded as described below (see also Additional file 2) and as detailed in [11].^2^ First, we compared all the HPO terms, which annotate all the D in a given PS, in order to retrieve, for *M* original terms, *M* representative terms annotating the PS. An intra-PS shared term is either an identical term or a common ancestor of different terms (here, the closest ancestor in the HPO hierarchy). The final representative term is the middle term in a list of common ancestors (arranged in ascending order of specificity). Second, for all the possible PS-PS pairs, we compared the representative HPO terms that annotate each PS in the pair, in order to retrieve the terms shared by both PS. An inter-PS shared term is either an identical term or a common ancestor of two different terms (here, the most informative common ancestor) annotating both PS in the pair. To define the most informative ancestor, an Information Content (IC) score according to Resnik had already been calculated [12] and assigned to each HPO term. Third, as each PS is annotated with multiple HPO terms, a similarity matrix containing all pairwise IC was assembled and a similarity coefficient was calculated according to the ‘best-match average’ strategy, meaning the average of all maximum similarities on each row and column of the matrix [13]. Finally, the CSN was assembled by joining the PS to each other and assigning, as weight *w* for each edge, the value of the similarity coefficient [14].

The same procedure was followed to assemble three additional networks of biological similarity (BSN-BP, BSN-CC and BSN-MF), except that we used the GO annotations of the genes, whose mutations cause the D in the PS under study. Besides generating one network for each of the three GO sub-ontologies, a general network of biological similarity (designated BSN) was generated, in which the weight *w* for each edge is the highest similarity coefficient among the three sub-ontology-related BSN. Specifically, the BSN-BP, BSN-CC and BSN-MF contributed respectively to 60.3, 30.2 and 9.5% of the edges in the general BSN. Cytoscape [15] and Network Analyzer [16] were used to display and analyze all the networks. To select the strongest PS-PS associations only, a threshold for the edge weights *w* was set, as described [17].

### Disease classification

Each node in the networks represents a PS and is color-coded according to the DO term that annotates the majority of D in that PS [2]. The DO terms used are the child terms of the root *Disease* (*Syndrome*, *Genetic disease*, *Physical disorder*, *Disease by infectious agent*, *Disease of metabolism*, *Disease of mental health*, *Disease of cellular proliferation* and *Disease of anatomical entity*). We also used the child terms of *Disease of anatomical entity*, which designate disorders of the cardiovascular, endocrine, gastrointestinal, hematopoietic, immune, integumentary, musculoskeletal, nervous, reproductive, respiratory, thoracic and urinary systems.

### Cluster analysis

For cluster analysis, the binary similarity coefficients between any PS_i_ and PS_j_ pair (from a set of *m* PS) were assembled in a symmetrical matrix *M*. The matrix is composed of *m* rows and *m* columns (each row and each column corresponding to one PS), so that *M*_*ij*_ denotes the (either HPO- or GO-based) similarity between the *i*^*th*^ and the *j*^*th*^ PS. Then, hierarchical cluster analysis was done using Multiple Experiment Viewer, with Pearson correlation uncentered (as distance metrics) and average-linked (as linkage method).

## Results

### The clinical similarity network (CSN)

To assemble the CSN, we first retrieved all the molecularly defined D that belong to the OMIM-encoded PS. Then, for each D, we retrieved all the HPO-encoded DP and identified all the shared DP for each PS pair. Finally, we assembled the CSN, by displaying each PS as a node and linking each pair of nodes with an edge. Each edge represents the clinical similarity of the two PS in the pair and has a weight *w*, proportional to their similarity coefficient (Additional files 1 and 2).

The CSN contains 293 nodes linked by 42,778 edges. This means the CSN is a fully connected network, in which each node is linked to all the other nodes (self-loops excluded), because each PS has some similarity to all the other PS. However, most edges only represent weak similarities, as any pair of HPO annotations (no matter how dissimilar) shares at least one ancestor of low specificity in the ontology (see Background). Nevertheless, by gradually raising the threshold for *w*, one can remove the weaker similarities, retaining only the stronger ones – the edges that link highly similar PS. Clearly, the removal of the edges also results in a loss of nodes. However, the threshold can be set appropriately, so that the maximal removal of edges is obtained with only minimal loss of nodes (Fig. S1*panel A*; Additional file 3).

Next, to analyze the topological properties of the CSN, we set a threshold of 1.0. Here, the majority (87%) of the less specific edges was removed, with just minimal loss (2%) of the nodes and no fragmentation of the network (Table 1). The CSN retained 287 nodes and 5660 edges and was still composed of one giant connected component (GCC). The first parameter we assessed was the connectivity *k*_*i*_, i.e., the number of direct neighbors of a given node *i*, which indicates how many other nodes node *i* is linked to (and thus how many PS are clinically similar to PS_*i*_). An average connectivity <*k*> of 39.4 indicates that, on average, each PS in the CSN is connected to 39 other PS with a similarity coefficient > 1.0. Interestingly, PS in different DO classes differ in their <*k*> (not shown), with the PS of the DO class *Syndrome* having the highest <*k*> (66 ± 33); this probably reflects the wide variety of DP that characterizes syndromic PS (and thus their larger chance of being similar to other PS).

**Table 1:** Network analysis

		Network				
**Threshold**	**Parameter**	**CSN**	**BSN-BP**	**BSN-CC**	**BSN-MF**	**BSN**
*0.0*	*Nodes*	293	316	314	305	319
	*Edges*	42,778	49,770	49,141	46,360	50,721
*1.0*	*Nodes*	287	298	258	209	311
	*Edges*	5660	11,223	2821	1633	13,282
	*<k>*	39.440	75.320	21.870	15.630	85.420
	*<C>*	0.635	0.708	0.653	0.691	0.654
	*<l>*	2.294	1.950	2.486	2.829	1.799
	*Density*	0.138	0.254	0.085	0.075	0.276

The topological properties of the five networks. Network analysis was conducted after setting a threshold of 1.0 for all the networks. At this threshold, all the networks are still composed of a single GCC, despite the loss of all the edges with weight *w* ≤ 1.0

Table 1 reports two additional parameters. The clustering coefficient *C*_*i*_ of node *i* is the ratio of the actual to the theoretically maximal number of edges that link mutually the neighbors of *i*. A <*C*> (i.e., the average *C* of all the nodes in the CSN) of 0.635 indicates that 63.5% of the PS, which are clinically similar to a given PS, are similar to each other as well. Finally, the shortest path *l*_*i,j*_ of a given pair of nodes *i* and *j* is the number of edges that one must travel to go from *i* to *j*. An average *l* (or <*l*>) of 2.29 (≈ 2) indicates that, even for those pairs of PS that are not similar to each other (and thus have *l* ≠ 1, because they are not linked directly in the CSN), there is at least a third PS that is similar to the two unconnected PS. Taken together, the high <*k*>, the high <*C*> and the low <*l*> indicate that, already at a threshold of 1.0, most PS have, on average, a significant degree of clinical similarity.

### Islands and clusters of high clinical similarity within the CSN

**Fig. 2:**
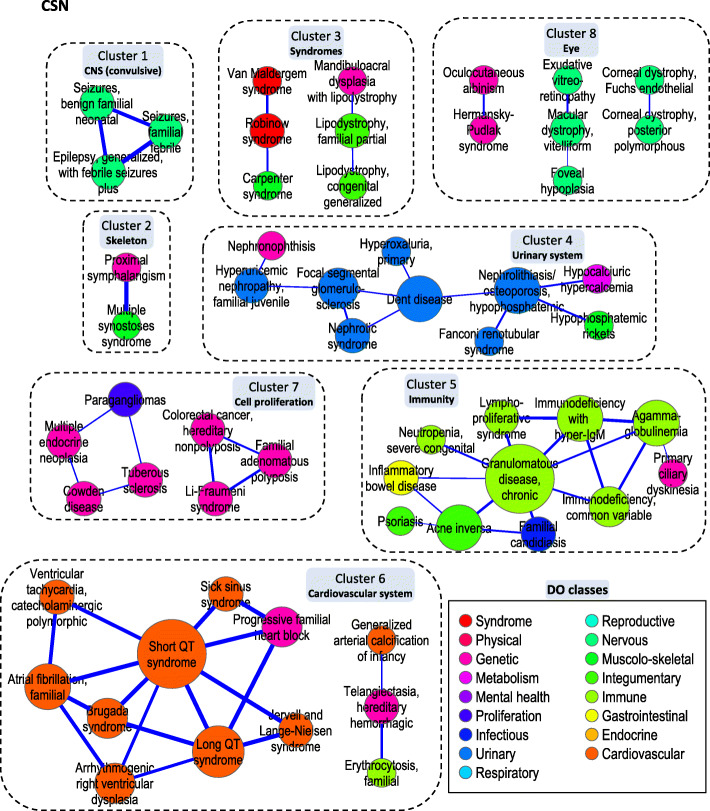
The CSN. The CSN, at a threshold of 2.46, contains 58 nodes linked by 63 edges and is fragmented into 13 islands and 8 clusters. *Node color* indicates the DO class (*inset*), while *node size* is proportional to the connectivity *k* of the PS. Edges represent biological similarity and *edge thickness* is proportional to the weight *w* (i.e., the maximal GO-based similarity among the three GO sub-ontologies)

In all the networks, to focus on groups of highly similar PS, we raised the threshold until 20% of the initial number of PS were retained. In the CSN, when the threshold was increased to 2.46, we retained 58 nodes (20% of the PS) and just 63 edges (0.2% of the PS-PS similarities). The CSN was fragmented into 13 islands, each composed of PS that were highly similar with respect to the clinical DP and, accordingly, to the clinical DO class as well (Fig. 2; *node colors*). The highest similarity links *Seizures, familial febrile* (PS121210) with *Epilepsy, generalized, with febrile seizures, plus* (PS604233). It exemplifies how the similarity coefficient reflects the sharing of highly informative DP, including both identical terms annotating the two PS (e.g., *Febrile seizures*) and common ancestors of different terms (e.g., *Dialeptic seizures*, which is the ancestor of both *Absence seizures* and *Focal seizures with impairment of consciousness or awareness*, in PS121210 and PS604233, respectively).

CSN fragmentation, however, does not necessarily imply that disconnected PS are dissimilar, but simply that the similarity coefficient falls below the chosen threshold. It remains possible that even disconnected PS share substantial similarity to each other (and dissimilarity from the other PS). To analyze this, we examined the inter-PS similarity by another approach, hierarchical cluster analysis. Within the CSN (still at the threshold of 2.46), we identified eight clusters, many of which were mostly composed of clinically similar PS islands (*dotted lines* in Fig. 2), like the convulsive PS of cluster 1. In addition, clustering allowed grouping similar PS that, at this threshold, are disconnected. For instance, seven PS scattered in three small islands could be grouped within cluster 8, which is characterized by ocular involvement (though affecting different components of the eye, such as iris, retina and cornea).

Finally, the similarity among the PS may extend beyond the boundaries of the clusters. For instance, *Inflammatory Bowel Disease* (PS266600; cluster 5) and *Colorectal cancer, hereditary non-polyposis* (PS120435; cluster 7) have a similarity coefficient of 2.17, which is 4.2 times the average similarity in the whole CSN (0.52 ± 0.72), but lower than the threshold of 2.46. Their clinical similarity is mostly due to the shared DP *Abnormality of the large intestin*e, which is the common ancestor of *Rectal abscess* (PS266600) and *Colon cancer* (PS120435).

### The biological similarity networks (BSN)

**Fig. 3:**
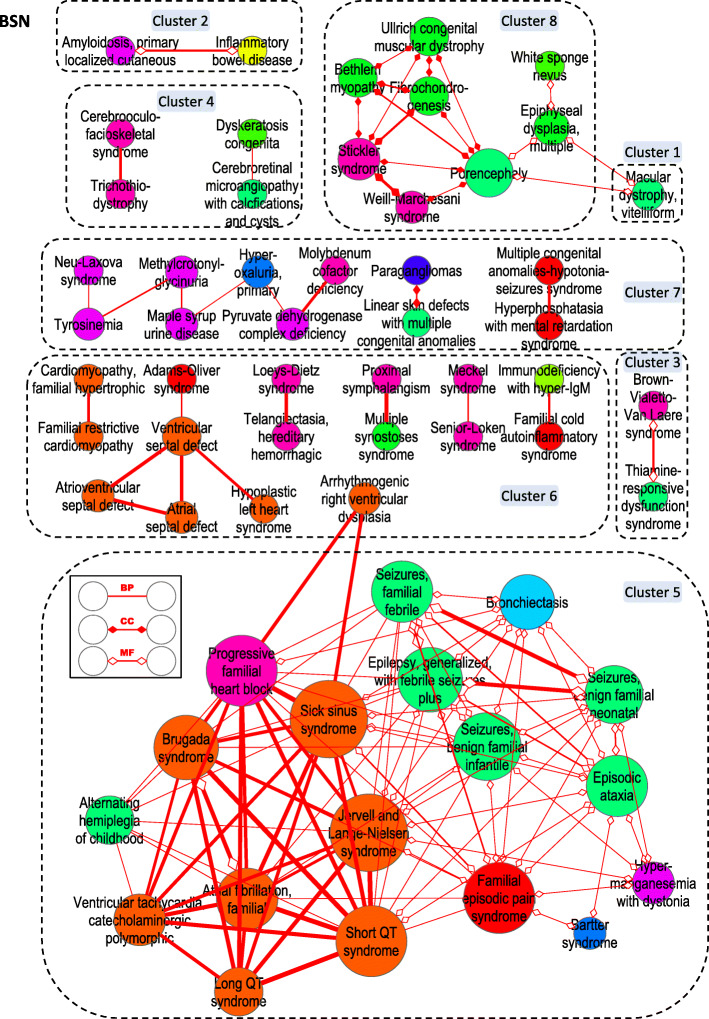
The BSN. The BSN, at a threshold of 2.70, contains 68 nodes linked by 138 edges and is fragmented into 18 islands and 8 clusters. *Node color* indicates the DO class (see Fig. 2, *inset*) and *node size* is proportional to the connectivity *k* of the PS. Edges represent clinical similarity and *edge thickness* is proportional to the weight *w* (i.e., the HPO-based clinical similarity)

To assemble the BSN we retrieved the following data and then proceeded similarly to the assembly of the CSN. First, for each PS-associated D we retrieved the corresponding DGP from OMIM. Second, for each DGP we retrieved the corresponding biological annotations from the BP, CC and MF sub-ontologies of GO. Third, we paired all the PS and identified which GO annotations were shared by each pair of PS. Finally, we assembled three networks (see Additional file 3), i.e., the BSN-BP (Fig. S2), BSN-CC (Fig. S3) and BSN-MF (Fig. S4), by linking each pair of PS with a weighted edge, which represents their degree of biological similarity within the relevant sub-ontology. We also assembled a more general network (the BSN), in which an edge (linking a given pair of PS) is the one with the highest *w* among the three edges that link the same PS pair in the three sub-ontology BSN (Fig. 3). Like the CSN, the BSN-BP, BSN-CC, BSN-MF and BSN are also densely connected networks, even though most edges represent weak similarities. Thus, to focus on the PS with the highest degree of similarity, we gradually raised the threshold for *w* (Fig. S1*panels B-E*).

As for the CSN, network analysis was done at a threshold of 1.0, where all four biological networks are still composed of one GCC, in spite of having lost many edges. However, the networks differ in the fractions of nodes and edges retained (compared to the initial no-threshold condition; Table 1). More edges are retained in the BSN-BP than in the BSN-CC or – even more – the BSN-MF, indicating that BP generates more informative edges than either CC or MF. In addition, compared to the BSN-CC and BSN-MF, the BSN-BP is a denser network, whose nodes have more connections and lie closer to each other. All these parameters are even more marked in the general BSN, which is not surprising, as each edge, which links two PS in the general BSN, is the most informative edge linking the same two PS in the three sub-ontology-related BSN.

### Islands and clusters of high biological similarity within the BSN

The threshold was further raised until about 20% of the PS were retained (Fig. S1*panels B-E*) and the networks were fragmented into islands of biologically similar PS. As for the CSN, hierarchical cluster analysis identified clusters of PS in the BSN-BP (Fig. S2), BSN-CC (Fig. S3), BSN-MF (Fig. S4) and in the general BSN (Fig. 3). Most clusters had a clear biological meaning, which can be expressed by the most significant GO terms that annotate the DGP in each cluster. We indicate these enriched GO terms as the labels of the clusters. Thus, most PS clusters in the BSN-CC can be interpreted as organelle D (e.g., ciliopathies), which are due to defects in a distinct sub-cellular structure (the cilium; Fig. S3, *cluster 7*).

In the largest cluster of the BSN (Fig. 3, *cluster 5*), the majority of PS are cardiac D (*orange nodes*), particularly, ion-channel disorders [18], which are characterized clinically by arrhythmias. The highest similarity coefficient links *Brugada syndrome* with *Short QT syndrome*, because these PS share numerous highly informative processes, including the BP-annotated *Potassium ion export from cell*. Surprisingly, however, other PS in the cluster are not cardiac but convulsive D (e.g., *Seizures, benign familial infantile*; *green*), as well as respiratory (*Bronchiectasis*; *light blue*), renal (*Bartter syndrome*; *blue*) and syndromic (*Familial episodic pain syndrome*; *red*) disorders. The same PS cluster together in the BSN-BP (Fig. S2, *cluster 8, Ion transmembrane transport*), BSN-CC (Fig. S3, *cluster 8, Ion channel complexes*) and the BSN-MF (Fig. S4, *cluster 5, Transmembrane transporter activities*). Thus, the BSN identifies clusters of biologically related (albeit clinically unrelated) PS.

### Correlating the clinical and the biological similarities

**Fig. 4:**
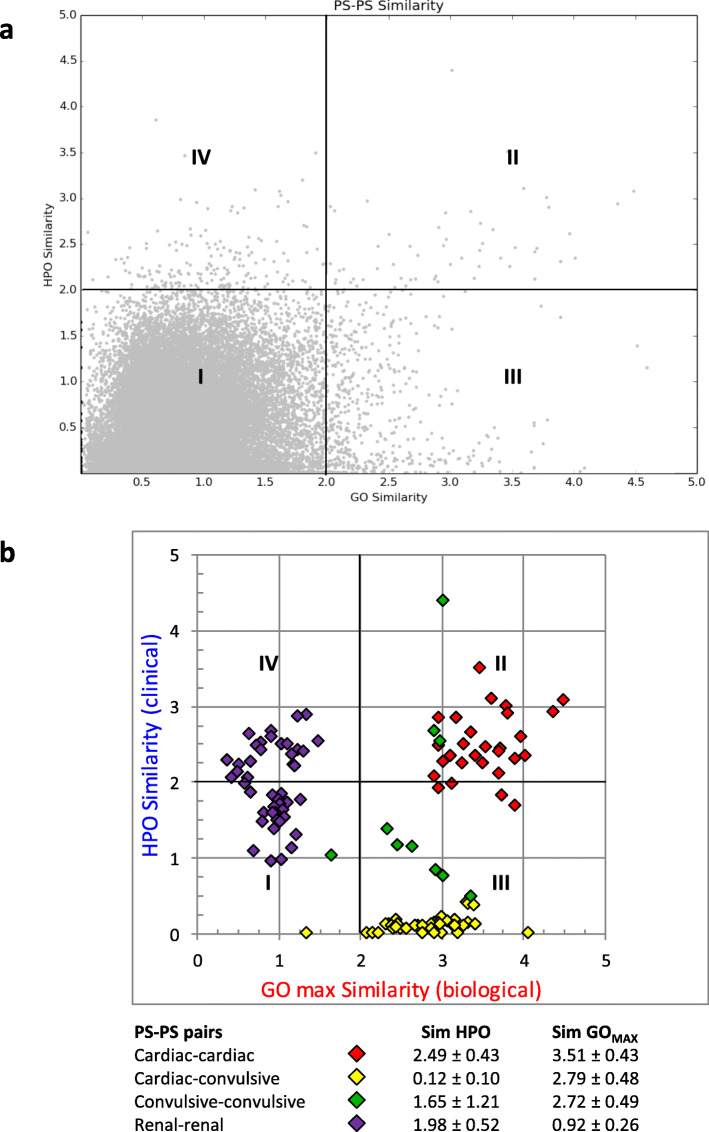
Comparing biological and clinical similarities. **a** The scatter plot displays the correlation between clinical and biological similarity (as defined in the CSN and the BSN, respectively). **b** Details of the subset of PS-PS discussed in the Results

Up to now, we have considered the clinical and biological similarity as separate entities. Next, to correlate the two, we assembled a scatter plot of the similarity coefficients from the CSN and the general BSN (Fig. 4a). The plot represents the 42,782 pairs of PS, for which both clinical and biological coefficients are available, and can be divided into four quadrants. First, *quadrant I* comprises the majority of PS pairs, which have low clinical and low biological similarity. Second, many PS pairs (*quadrant II*) have high clinical and high biological similarity (both coefficients being ≥ 2 in 57 PS pairs). For example, many of the ion transport-related PS involving cardiac arrhythmias have close similarity in both clinical and biological terms (Fig. 4b, *red diamonds*). Likewise, a subset of the ion transport-related PS with a convulsive DP have medium-high levels of similarity, both clinical and biological (Fig. 4b, *green diamonds*).

Third, many pairs (*quadrant III*) have high biological but low clinical similarity, the similarity coefficients being ≥ 2 (biological) and < 2 (clinical) in 500 pairs. Examples include many of the clinically heterogeneous pairs of ion transport-related PS discussed above, which are composed of one cardiac arrhythmia PS and one convulsion-related PS (Fig. 4b, *yellow diamonds* and *orange/green nodes* in Fig. 3, cluster 5).

Fourth, many PS pairs (*quadrant IV*) have high clinical and low biological similarity, with similarity coefficients ≥ 2 (clinical) and < 2 (biological) in 178 PS pairs. The renal D in the CSN (Fig. 4b, *purple diamonds* and *blue nodes* in Fig. 2, cluster 4) reflect, on the clinical side, the sharing of highly informative clinical DP (e.g., *Proximal tubule nephrolithiasis* or *Bone pain and fractures*). On the biological side, however, they involve alterations in biological functions as dissimilar as *Endocytosis* (*Dent’s disease*) [19], *Glyoxylate metabolism* (*Primary hyperoxaluria*) [20] and *Absorption of phosphate* (*Nephrolithiasis hypophosphatemic*) [21]. Taken together, these observations reveal widely different types of correlation between the clinical phenotypes of the D and the biological functions of the corresponding DGP.

## Discussion

The main findings of this study are that, first, a series of weighted networks allowed displaying the clinical and biological similarity of the PS that are composed of molecularly-characterized D. Second, raising the similarity threshold allowed identifying subsets of PS with progressively higher degrees of clinical and/or biological similarity. Third, there were several examples of PS pairs, whose clinical and biological similarities are correlated either directly or inversely.

The use of networks in medicine [7, 9] and pharmacology [22, 23] for diverse purposes is not unprecedented, including the investigation of similarity among D [5, 6, 8]. The novelty of our study, however, consists of basing the analysis not on individual D but on the PS [3], which can be regarded as meta-nodes of the networks. The use of PS is justified by the wide numbers of locus heterogeneity-like conditions in human genetics, where mutations in different genes cause similar D that are hardly – if at all – distinguishable clinically. Thus, by merging similar D into the same PS (or, in graphical terms, by absorbing thousands of D nodes into hundreds of PS meta-nodes), the PS greatly simplified our analysis of similarity. An alternative approach (focused on the D) would have required comparing millions of D-associated DP and then identifying clusters of similar D. Thus, the use of the PS is advantageous, because it relies on authoritative clinical judgment (and not on the arbitrary choice of a similarity threshold) to define the boundaries of clinically similar D clusters.

In addition to the OMIM-defined PS, the study also benefited from the availability of metrics that quantify the similarity of both the clinical (HPO-defined) annotations [5] and the biological (GO-defined) ones [13], which are shared by each pair of PS. Both HPO and GO are ontologies, in which each term is a specific instance of a more general parent term. Thus, when comparing two annotations, the similarity-searching methods identify which terms are shared within the hierarchical structure of the ontology. Consequently, many shared terms are common ancestors that have various levels of IC. At the lowest extreme, even the most dissimilar annotations share, as common ancestor, the root, which is the least specific term of the ontology (and thus, the term with the lowest IC). The downside of defining similarity based on common ancestry is that, at the end of the procedure, each PS has some similarity with all the other PS. In addition, the resulting network has limited usefulness, because each of its *n* nodes is connected to all the other (*n*-1) nodes by means of all the possible n*(*n*-1)/2 edges. Nevertheless, assigning a similarity coefficient to each PS pair allows converting the fully-connected network into a weighted graph [14, 24], in which the weight of each edge is directly proportional to the strength of the similarity. Then, by raising the threshold for the weight, it becomes feasible to retain only the strongest links, making the network more usable for analytical purposes. Even though the loss of edges comes inevitably at the expense of a progressive fragmentation of the network, the resulting fragments are indeed the subsets of highly similar PS that were the initial goal of the study.

Far from considering the clinical and biological similarities as separate entities, this study also set out to correlate them. One of the basic questions at the inception of the study was to test whether the altered biological functions, due to gene mutations (at the level of cells and tissues), might account for the clinical phenotypes in the patient (at the level of organs and systems). We had hypothesized that the clinical and biological similarities should be directly correlated, so that clinically similar PS should be biologically similar as well. We did identify PS that were similar not only clinically (in the CSN) but also biologically (in the BSN). For example, similar clusters of cardiac arrhythmia PS were detectable in both CSN and BSN. However, as the scatter plot indicates, correlations of the two types of similarity are more complex than expected. Clearly, in many instances missing clinical-biological correlations are merely due to non-specific annotations of the DP (in HPO) and/or the DGP (in GO). In particular, in a binary PS-PS comparison, even a low specificity annotation of just one of the two PS (and in just one of the two ontologies) means a poorly informative common ancestor is retrieved. This, in turn, lowers the overall similarity coefficient of the two PS (with regard to that ontology), ultimately affecting the correlation of the clinical and biological similarity. Nevertheless, we propose that several instances of missing correlation reflect not simply defective annotations but conditions of potential pathogenic interest. For ease of analysis, we identified two major conditions where the biological and clinical similarities are not directly correlated.

The first condition consists of PS pairs with high biological, but low clinical, similarity. The category is exemplified by the PS, whose normal – non-mutated – gene products participate in the channel-mediated transport of cations during the repolarization of excitable cells. However, in spite of this high degree of biological similarity, the PS comprise clinical conditions as diverse as cardiac arrhythmia- and seizure-related disorders. A likely explanation for the divergent clinical manifestations of a similar biological dysfunction is the tissue-restricted expression of many DGP. For instance, several ion channel subunits are specifically expressed in excitable cells of either the heart or the brain [25]. In addition, our analysis shows that these PS, in turn, are part of a wider family of D, the ‘channellopathies’, whose normal gene products participate in transporting ions across the membranes, at the level of voltage-gated (cardiac, convulsive and episodic pain-related disorders) and ligand-gated (bronchiectasis and Bartter syndrome) ion channels. Because in the GO database a given MF (such as transporter activity) takes place in a defined CC (an ion channel) to ensure a specific BP (in this case, ion transport), the three sub-ontology-related BSN converge in identifying communities of clinically diverse, but biologically related, disorders.

The second condition consists of PS pairs with high clinical, but low biological, similarity. We propose that this category illustrates how different biological mechanisms converge in a composite response, resulting in clinically similar DP. An interesting example is the cluster of proximal tubule nephrolithiasis, discussed in the Results section (Fig. 2, cluster 4). Another example is a cluster of three PS in the CSN (van Maldergem, Robinow and Carpenter syndromes), which all cause severe skeletal dysplasia with craniofacial anomalies (Fig. 2, cluster 3). On the one hand, the corresponding DGP annotate apparently unrelated BP, accounting for their low biological similarity. On the other, however, most of the DGP (WNT5A, DVL1, DVL3 and ROR2 in Robinow syndrome, FAT4 and DCHS1 in van Maldergem syndrome and RAB23 in Carpenter syndrome) participate in the non-canonical Wnt signaling pathway and in the process of planar cell polarity, which are key regulators of embryonic development [26].

## Conclusions

In conclusion, our findings indicate that, even with the intrinsic limitations of the available databases and current biological understanding, it is already possible to rely on semi-automated procedures to identify altered biological responses as likely mechanisms for many inherited D with a known molecular basis.

## Supplementary information

**Additional file 1.** Definitions of the terms used in the study

**Additional file 2.** Explanation of the algorithm used to generate the similarity networks

**Additional file 3: Fig. S1.** Network fragmentation. Increasing the similarity threshold in the CSN (*A*), BSN-BP (*B*), BSN-CC (*C*), BSN-MF (*D*) and the general BSN (*E*) progressively reduces the fraction of nodes (*open diamonds*) and edges (*closed diamonds*), with a consequent fragmentation of the initial network. Results are shown as fractions of the total numbers of nodes and edges in the whole networks (i.e., at a similarity threshold of zero). Also shown is the threshold of 1.0 (*vertical dotted lines*) used for network analysis and the thresholds (*vertical dashed lines*) used to retain 20% of the initial nodes (*horizontal dotted lines*) and to display the networks in Figs. 2 (CSN), S2 (BSN-BP), S3 (BSN-CC), S4 (BSN-MF) and 3 (general BSN). **Fig. S2.** The BSN-BP. The BSN-BP, shown at a threshold of 2.5, contains 67 nodes linked by 135 edges and is fragmented into 14 islands and 9 clusters. In Figs. S2, S3 and S4, *node color* indicates the DO class (see *inset* of Fig. 2), while *edge thickness* is proportional to the weight *w* (i.e., the degree of HPO-based clinical similarity between PS). **Fig. S3.** The BSN-CC. The BSN-CC, shown at a threshold of 1.9, contains 63 nodes linked by 93 edges and is fragmented into 9 islands and 8 clusters. **Fig. S4.** *The BSN-MF.* The BSN-MF, shown at a threshold of 2.2, contains 58 nodes linked by 154 edges and is fragmented into 11 islands and 9 clusters.

## Data Availability

All the datasets used in this manuscript are publicly available from the following sources: OMIM (https://www.omim.org/downloads/) for the gene map (genemap2.txt) and the morbid map (morbidmap.txt), which can be accessed upon registration without a license. The full list of the PS is available from OMIM on request. HPO (http://www.obofoundry.org/ontology/hp.html) for the DP that annotate the D, i.e. the dataset phenotype_annotation.tab available at. http://compbio.charite.de/jenkins/job/hpo.annotations/lastStableBuild/artifact/misc/phenotype_annotation.tab . DO (http://www.obofoundry.org/ontology/doid.html) for the etiological classification of the D, as detailed in the doid.obo dataset at http://purl.obolibrary.org/obo/doid.obo . GO (http://geneontology.org/) for the biological annotations of the DGP (in the *Homo sapiens* EBI Gene Ontology Annotation Database available at. ftp://ftp.ebi.ac.uk/pub/databases/GO/goa/HUMAN/goa_human.gaf.gz). The key data generated during the analysis of the above datasets are included in this published article and its additional files. The scripts, as well as the complete datasets for assembling the networks, are available at https://github.com/alessio-gamba/Similarity_Inter_PS .

## Abbreviations

BP: Biological Process
BSN: Biological Similarity Network
CC: Cellular Component
CSN: Clinical Similarity Network
D: Diseases
DGP: Disease Gene Product
DO: Disease Ontology
DP: Disease Phenotype
GCC: Giant Connected Component
GO: Gene Ontology
HPO: Human Phenotype Ontology
IC: Information Content
MF: Molecular Function
OMIM: Online Mendelian Inheritance in Man
PS: Phenotypic Series

## Footnotes

1 Gamba A, Salmona M, Bazzoni G. *The Similarity of Inherited Diseases (I): Clinical Similarity within the Phenotypic Series.* 2019. (Under review)

2 For a complete description of the procedure, see *Gamba A. Approaches to the molecular basis of genetic diseases (PhD thesis).* Preprint at https://air.unimi.it/retrieve/handle/2434/545159/947802/phd_unimi_R11022.pdf

## References

[1] Köhler S, Vasilevsky NA, Engelstad M, Foster E, McMurry J, Aymé S (2017). The human phenotype ontology in 2017. Nucleic Acids Res.

[2] Kibbe WA, Arze C, Felix V, Mitraka E, Bolton E, Fu G (2015). Disease ontology 2015 update: an expanded and updated database of human diseases for linking biomedical knowledge through disease data. Nucleic Acids Res.

[3] Amberger JS, Bocchini CA, Schiettecatte F, Scott AF, Hamosh A (2015). OMIM.org: online Mendelian inheritance in man (OMIM®), an online catalog of human genes and genetic disorders. Nucleic Acids Res.

[4] Ashburner M, Ball CA, Blake JA, Botstein D, Butler H, Cherry JM (2000). Gene ontology: tool for the unification of biology. The Gene Ontology Consortium. Nat Genet.

[5] Hoehndorf R, Schofield PN, Gkoutos GV (2015). Analysis of the human diseasome using phenotype similarity between common, genetic, and infectious diseases. Sci Rep.

[6] Menche J, Sharma A, Kitsak M, Ghiassian SD, Vidal M, Loscalzo J (2015). Disease networks. Uncovering disease-disease relationships through the incomplete interactome. Science.

[7] Vidal M, Cusick ME, Barabási A-L (2011). Interactome networks and human disease. Cell..

[8] Zhou X, Menche J, Barabási A-L, Sharma A (2014). Human symptoms-disease network. Nat Commun.

[9] Barabási A-L, Gulbahce N, Loscalzo J (2011). Network medicine: a network-based approach to human disease. Nat Rev Genet.

[10] Gene Ontology Consortium (2015). Gene ontology consortium: going forward. Nucleic Acids Res.

[11] Gamba A. Approaches to the molecular basis of genetic diseases. University of Milan (Italy); 2018.

[12] Köhler S, Schulz MH, Krawitz P, Bauer S, Dölken S, Ott CE (2009). Clinical diagnostics in human genetics with semantic similarity searches in ontologies. Am J Hum Genet.

[13] Wang JZ, Du Z, Payattakool R, Yu PS, Chen C-F (2007). A new method to measure the semantic similarity of GO terms. Bioinforma Oxf Engl.

[14] Barrat A, Barthélemy M, Pastor-Satorras R, Vespignani A (2004). The architecture of complex weighted networks. Proc Natl Acad Sci U S A.

[15] Yeung N, Cline MS, Kuchinsky A, Smoot ME, Bader GD (2008). Exploring biological networks with Cytoscape software. Curr Protoc Bioinformatics.

[16] Assenov Y, Ramírez F, Schelhorn S-E, Lengauer T, Albrecht M (2008). Computing topological parameters of biological networks. Bioinforma Oxf Engl.

[17] Hidalgo CA, Blumm N, Barabási A-L, Christakis NA (2009). A dynamic network approach for the study of human phenotypes. PLoS Comput Biol.

[18] Cerrone M, Napolitano C, Priori SG (2012). Genetics of ion-channel disorders. Curr Opin Cardiol.

[19] Devuyst O, Thakker RV (2010). Dent’s disease. Orphanet J Rare Dis.

[20] Cochat P, Rumsby G (2013). Primary hyperoxaluria. N Engl J Med.

[21] Sayer JA (2017). Progress in understanding the genetics of calcium-containing nephrolithiasis. J Am Soc Nephrol JASN.

[22] Bazzoni G, Marengoni A, Tettamanti M, Franchi C, Pasina L, Djade CD (2015). The drug prescription network: a system-level view of drug co-prescription in community-dwelling elderly people. Rejuvenation Res.

[23] Hopkins AL (2008). Network pharmacology: the next paradigm in drug discovery. Nat Chem Biol.

[24] Newman ME. Scientific collaboration networks. II. Shortest paths, weighted networks, and centrality. Phys Rev E Stat Nonlinear Soft Matter Phys. 2001;(1 Pt 2):016132. 10.1103/PhysRevE.64.016132 11461356

[25] Seitter H, Koschak A. Relevance of tissue specific subunit expression in channelopathies. Neuropharmacology. 2017. 10.1016/j.neuropharm.2017.06.029 PMC7610580 28669898

[26] Butler MT, Wallingford JB (2017). Planar cell polarity in development and disease. Nat Rev Mol Cell Biol.

