# Supplementary material for: The similarity of inherited diseases (II): clinical and biological similarity between the phenotypic series
Source: BMC Med Genomics. 2020 Sep 24;13:139. doi: 10.1186/s12920-020-00793-y (PMC7513283; doi:10.1186/s12920-020-00793-y)
Supplement: Supplementary file 1 — Additional file 1. Definitions of the terms used in the study [file 12920_2020_793_MOESM1_ESM.docx]

**Additional File 1.** *Definitions of the terms used in the study*

| **Term** | **Definition** |
| --- | --- |
| *Phenotypic Series (PS)* | An OMIM-defined subset of genetic D that are clinically similar in spite of being caused by mutations of different genes (i.e., different DGP). |
| *Network* | A graph composed of nodes linked by edges. In the present study, nodes represent PS and edges indicate inter-PS similarities. In a weighted network, each edge has a weight that is proportional to a parameter (here, the Similarity Coefficient). |
| *Clinical Similarity Network (CSN)* | A weighted network, in which each node represents a PS, while each edge (linking two nodes) indicates the *clinical* similarity between the two PS. Clinical similarity is defined in terms of the HPO-derived DP that annotate the D in the PS. |
| *Biological Similarity Networks (BSN)* | A weighted network, in which each node represents a PS, while each edge (linking two nodes) indicates the *biological* similarity between the two PS. Biological similarity is defined in terms of the GO-derived annotations of the DGP that cause the D in the PS.  Three weighted BSN (BSN-BP, BSN-CC and BSN-MF) have been generated, each of which is derived from one of the three sub-ontologies of GO. In addition, a general BSN has been assembled. In the general BSN, the weight of the edge linking a given pair of PS is proportional to the greatest Similarity Coefficient among the three sub-ontology BSN. |
| *Information Content (IC)* | The amount of information of a given HPO or GO term. The IC value is the negative logarithm of the frequency of that term in its own (HPO or GO) database. |
| *Similarity Coefficient* | The Similarity Coefficient measures the (clinical or biological) similarity for a given pair of PS. Specifically, for each PS-PS pair, a similarity matrix is assembled that reports all the shared (clinical or biological) terms and their corresponding IC values. The Similarity Coefficient is the average of all the maximal IC on each row and column of the matrix. |
