# Supplementary material for: The similarity of inherited diseases (II): clinical and biological similarity between the phenotypic series
Source: BMC Med Genomics. 2020 Sep 24;13:139. doi: 10.1186/s12920-020-00793-y (PMC7513283; doi:10.1186/s12920-020-00793-y)
Supplement: Supplementary file 2 — Additional file 2. Explanation of the algorithm used to generate the similarity networks [file 12920_2020_793_MOESM2_ESM.docx]

**Additional File 2.docx** *Generation of a similarity network*

As mentioned in the main text, we followed the same method to define the clinical and biological similarity of the PS and to generate the corresponding similarity network. Thus, by *annotation* we refer to the term in the ontology that annotates either a clinical or a biological *object*. Specifically, the annotation is either a DP in HPO that annotates a D (as clinical object) or a term in GO that annotates a DGP (as biological object).

**Step 1: Defining the representative annotations of each PS**

Each PS is composed of objects and annotations. Ideally, it should be possible to associate each PS with the most specific terms that annotate all the objects in the PS. However, this is often not the case, because the objects in the same PS have annotations that differ not only in number but also in specificity (specificity being directly proportional to the distance from the root of the ontology). Thus, the aim of step 1 is to associate each PS with its *representative annotations*, i.e., terms providing not only a detailed but also a comprehensive description of that PS. In particular, care must be taken not to overestimate highly specific terms that annotate just one object – or a tiny minority of objects – in the PS. To this aim, we considered a term to be representative of a PS, only if it annotates at least half of its objects. The representative term can be either the one that directly annotates the objects in the ontology or a closest common ancestor shared by different terms.

*Inputs*

- A complete list of the PS (with all the objects and the annotations).

Each PS is composed of *n* objects (*o_1_*, *o_2_*, …, *o_n_*), each of which has *m* annotations (*a_1_*, *a_2_*, …, *a_m_*) and thus a total of *M* annotations:

$$M = \sum_{1}^{n} \left[ \left( a_{1},o_{1} \right),\left( a_{2},o_{1} \right),\cdots,\left( a_{m},o_{1} \right) \right], \left[ \left( a_{1},o_{2} \right),\left( a_{2},o_{2} \right),\cdots,\left( a_{m},o_{2} \right) \right] , \cdots\left[ \left( a_{1},o_{n} \right),\left( a_{2},o_{n} \right),\cdots,\left( a_{m},o_{n} \right) \right]$$

Note that the number *n* of objects varies among different PS and, likewise, the number *m* of annotations varies among different objects of the same PS.

By (*a_i_*, *o_j_*), we designate the *i^th^* annotation of the *j^th^* object in the PS.

- The ontology, with all the annotations and their hierarchical relations (e.g., ‘is a’ or ‘part of’).

*I/O*

1. Select the first PS
2. Select (*a_1_*, *o_1_*), i.e., the first annotation (*a_1_*) of the first object (*o_1_*) in the first PS
   1. Select all the *m* annotations of the first object [(*a_1_*, *a_2_*, …, *a_m_*), *o_1_*]
   2. Find the closest ancestor common to (*a_1_*, *o_1_*) and [(*a_1_*, *a_2_*, …, *a_m_*), *o_1_*].
   3. Go to 2.1 and select all the *m* annotations of the second object [(*a_1_*, *a_2_*, …, *a_m_*), *o_2_*].

Repeat steps 2.1 through 2.3 to compare (*a_1_*, *o_1_*) with all the *M* annotations of the PS

1. Sort in ascending order of specificity all the closest common ancestors (from steps 2.2)
2. Select the annotation in the middle of the sequence of ancestors (from step 3).

If the number of annotations *M* is odd, select the [(*M*+1)/2]*^th^* annotation (e.g., the 3^rd^ out of 5).

Else, if *M* is even, select the (*M*/2)*^th^* annotation (e.g., the 2^nd^ out of 4)

1. Assign to the PS the annotation from 4 as its first representative annotation
2. Go to 2 and repeat steps 2 through 5 for (*a_2_*, *o_1_*), i.e., the second annotation of *o_1_* in the first PS.

Repeat steps 2 through 6 for all the remaining *M* annotations of the first PS

1. Go to 1 and repeat steps 1 through 6 for all the remaining PS

*Output*

- A list of all the PS, each with its representative annotations.

**Step 2: Finding which representative annotations are shared by each pair of PS**

The aim of step 2 is to identify which representative annotations (identified in step 1) are shared by each possible *PS_i_-PS_j_* pair (*i* ≠ *j*). To this purpose, we compared (in a pairwise manner) all the representative annotations of *PS_i_* with those of *PS_j_*. A shared term is either an identical term or the most informative common ancestor of two different terms (i.e., the one with the highest IC among all the shared ancestors).

*Inputs*

- The list of all the representative annotations annotating each PS (i.e., the output of step 1). Here, let us assume that the *i^th^* and the *j^th^* PS have *m* and *n* representative annotations, respectively.
- The ontology.

*I/O*

1. Select a PS_i_-PS_j_ pair with their *m* and *n* representative annotations
2. Assemble an *m***n* similarity matrix reporting the *m* and *n* representative annotations of PS_i_ and PS_j_ as row and column headers, respectively.
3. Compare *a_i_* with *a_j_*, i.e., the *i^th^* with the *j^th^* representative annotations of the two PS.

If (*a_i_ = a_j_*), select *a_i_*.

Else, select the *most informative common ancestor* of *a_i_* and *a_j_*.

1. Repeat steps 1 through 3 for all the remaining pairs of PS.

*Output*

- All the shared representative annotations for each pair of PS.

**Step 3: Calculating the similarity coefficients for each pair of PS (‘best-match average’ strategy)**

With the aim of calculating a similarity coefficient for each *PS_i_-PS_j_* pair, in step 3 we first retrieve each similarity matrix (from step 2). Then, we attribute an IC to each shared representative annotation in each cell of the matrix and finally calculate the average of all the maximum IC on each row and column of the matrix.

*Inputs*

- All the *m***n* similarity matrices (from step 2), i.e., one matrix for each PS_i_-PS_j_ pair.
- The list of all the IC for each annotation in the ontology.

*I/O*

1. For each pair of PS, assemble a new *m***n* similarity matrix reporting the *m***n* IC of the corresponding representative annotations shared by the former and latter PS .
2. Retrieve the highest IC values in each row and each column
3. Calculate the mean value of the values in 2.

*Output*

- The list of all the PS-PS pairs with their Similarity Coefficients. The output – in the format '*PS_i_-(coefficient)_i,j_‑PS_j_*’ – is the tabular input for a weighted similarity network (with the PS representing the nodes and the coefficients the weighted edges).
