# Supplementary material for: The similarity of inherited diseases (II): clinical and biological similarity between the phenotypic series
Source: BMC Med Genomics. 2020 Sep 24;13:139. doi: 10.1186/s12920-020-00793-y (PMC7513283; doi:10.1186/s12920-020-00793-y)
Supplement: Supplementary file 3 — Additional file 3: Fig. S1. Network fragmentation. Increasing the similarity threshold in the CSN (A), BSN-BP (B), BSN-CC (C), BSN-MF (D) and the general BSN (E) progressively reduces the fraction of nodes (open diamonds) and edges (closed diamonds), with a consequent fragmentation of the initial network. Results are shown as fractions of the total numbers of nodes and edges in the whole networks (i.e., at a similarity threshold of zero). Also shown is the threshold of 1.0 (vertical dotted lines) used for network analysis and the thresholds (vertical dashed lines) used to retain 20% of the initial nodes (horizontal dotted lines) and to display the networks in Figs. 2 (CSN), S2 (BSN-BP), S3 (BSN-CC), S4 (BSN-MF) and 3 (general BSN). Fig. S2. The BSN-BP. The BSN-BP, shown at a threshold of 2.5, contains 67 nodes linked by 135 edges and is fragmented into 14 islands and 9 clusters. In Figs. S2, S3 and S4, node color indicates the DO class (see inset of Fig. 2), while edge thickness is proportional to the weight w (i.e., the degree of HPO-based clinical similarity between PS). Fig. S3. The BSN-CC. The BSN-CC, shown at a threshold of 1.9, contains 63 nodes linked by 93 edges and is fragmented into 9 islands and 8 clusters. Fig. S4. The BSN-MF. The BSN-MF, shown at a threshold of 2.2, contains 58 nodes linked by 154 edges and is fragmented into 11 islands and 9 clusters. [file 12920_2020_793_MOESM3_ESM.pptx]

## Slide 1
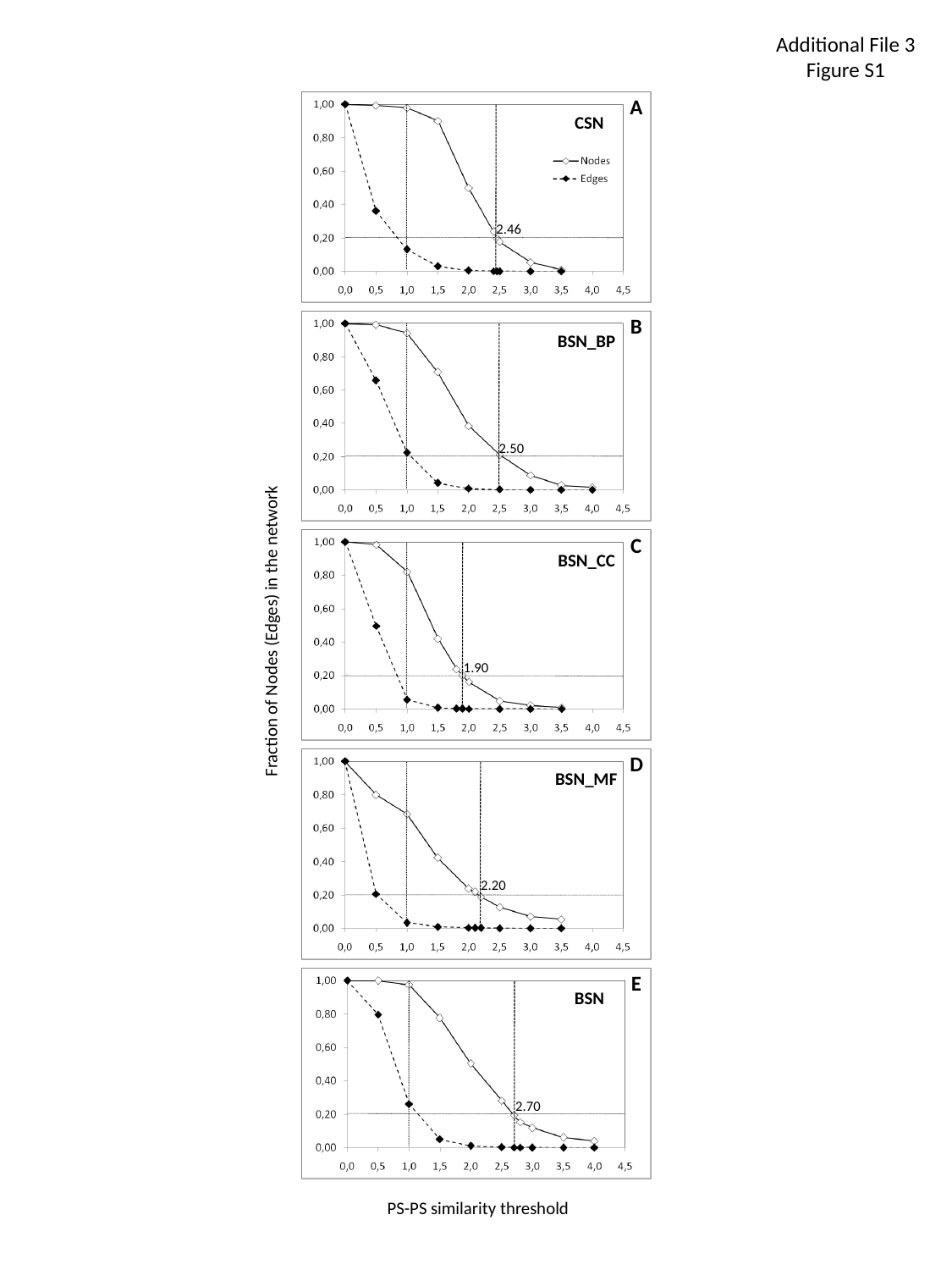

Additional File 3
Figure S1
A
CSN
2.46
B
BSN_BP
2.50
C
BSN_CC
1.90
D
BSN_MF
2.20
E
BSN
2.70
 Fraction of Nodes (Edges) in the network
PS-PS similarity threshold

## Slide 2
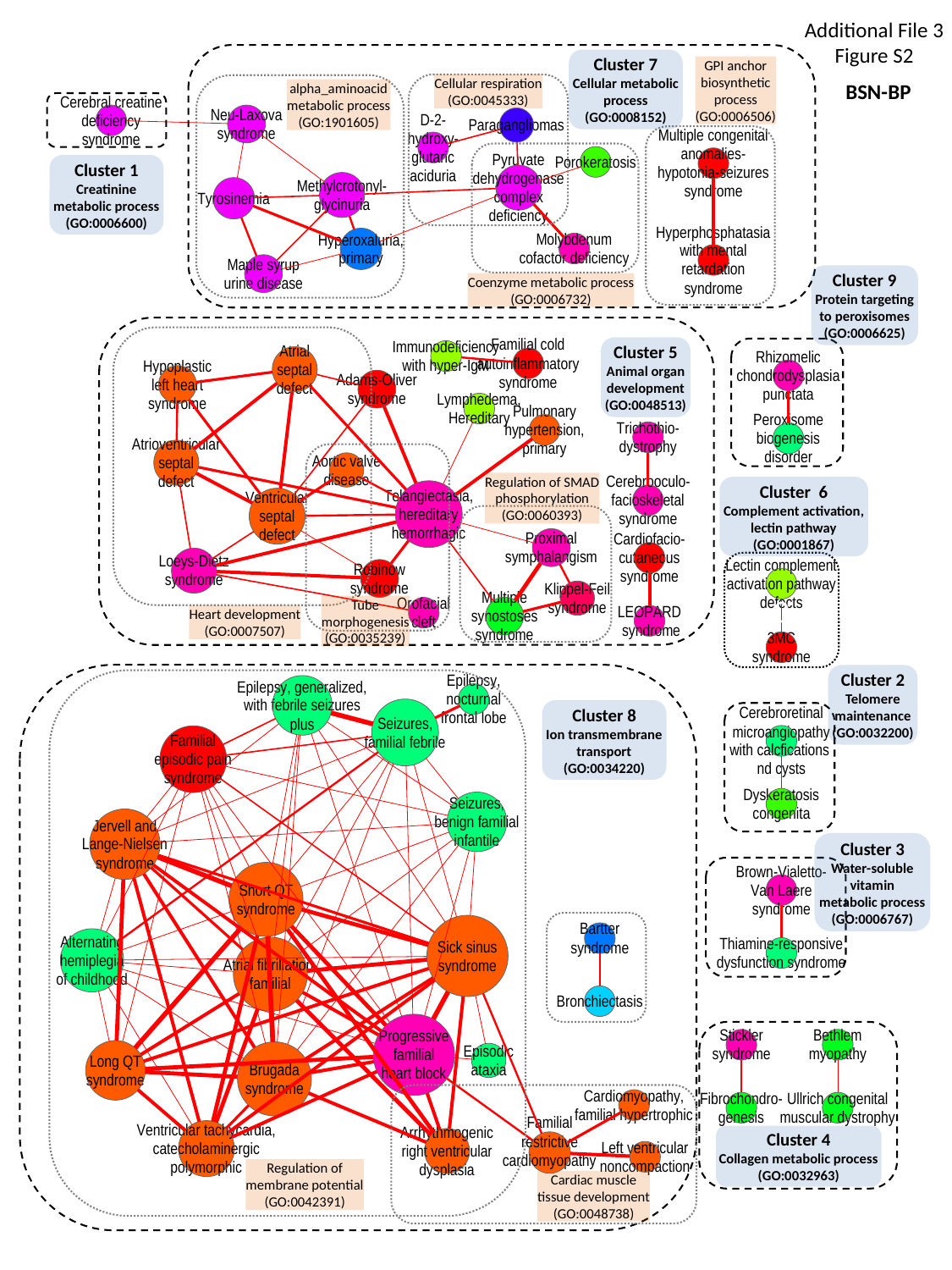

Additional File 3
Figure S2
Cluster 7
Cellular metabolic
process
(GO:0008152)
GPI anchor
biosynthetic
process
(GO:0006506)
BSN-BP
Cellular respiration
(GO:0045333)
alpha_aminoacid
metabolic process
(GO:1901605)
Cluster 1
Creatinine
metabolic process
(GO:0006600)
Cluster 9
Protein targeting
to peroxisomes
(GO:0006625)
Coenzyme metabolic process
(GO:0006732)
Cluster 5
Animal organ
development
(GO:0048513)
Regulation of SMAD
phosphorylation
(GO:0060393)
Cluster 6
Complement activation,
lectin pathway
(GO:0001867)
0
Tube
morphogenesis
(GO:0035239)
Heart development
(GO:0007507)
Cluster 2
Telomere
maintenance
(GO:0032200)
Cluster 8
Ion transmembrane
transport
(GO:0034220)
Cluster 3
Water-soluble
vitamin
metabolic process
(GO:0006767)
Cluster 4
Collagen metabolic process
(GO:0032963)
Regulation of
membrane potential
(GO:0042391)
Cardiac muscle
tissue development
(GO:0048738)

## Slide 3
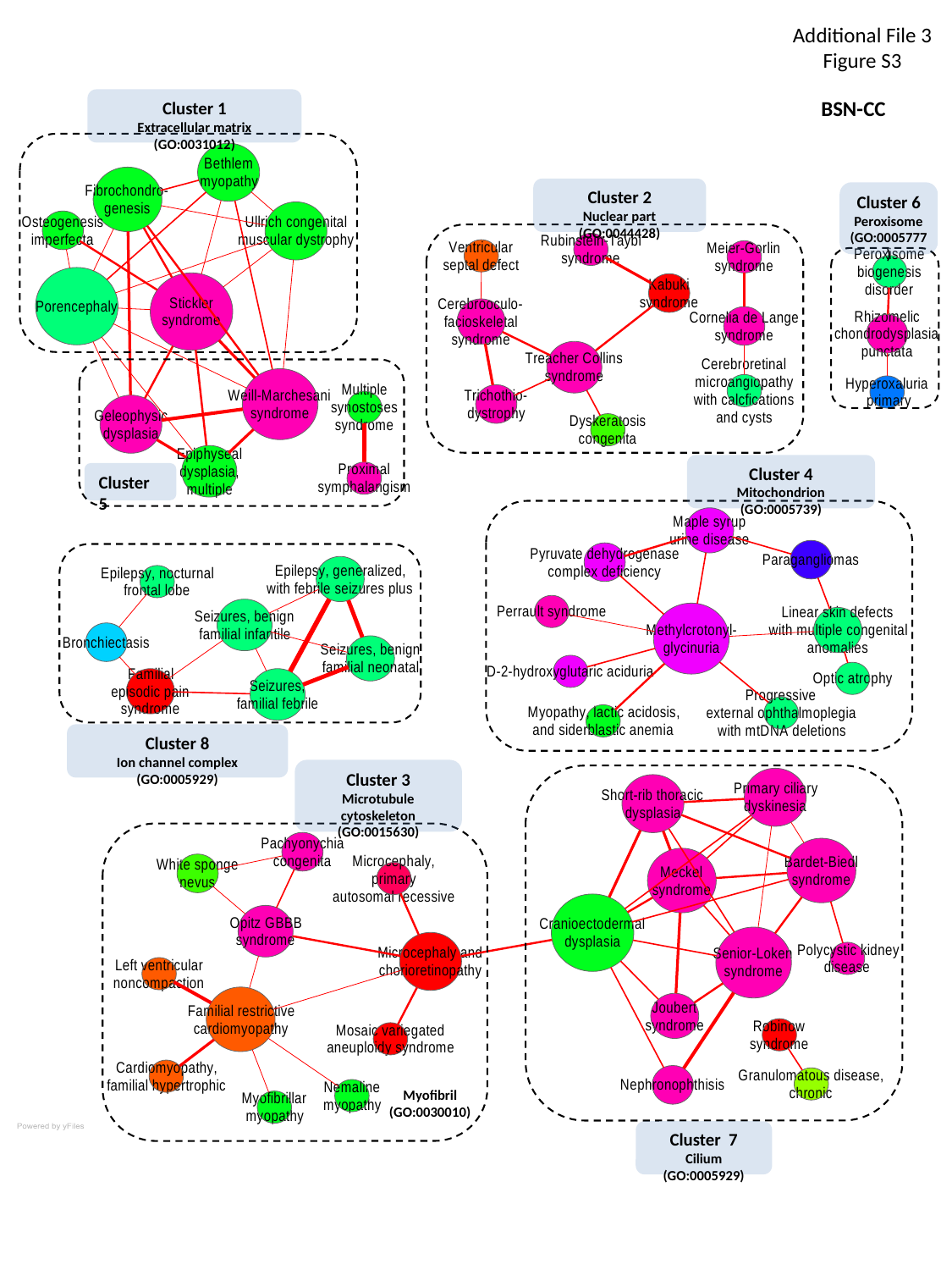

Additional File 3
Figure S3
Cluster 1
Extracellular matrix (GO:0031012)
BSN-CC
Cluster 2
Nuclear part (GO:0044428)
Cluster 6
Peroxisome
(GO:0005777)
Cluster 4
Mitochondrion (GO:0005739)
Cluster 5
Cluster 8
Ion channel complex (GO:0005929)
Cluster 3
Microtubule cytoskeleton
(GO:0015630)
Myofibril
(GO:0030010)
Cluster 7
Cilium (GO:0005929)

## Slide 4
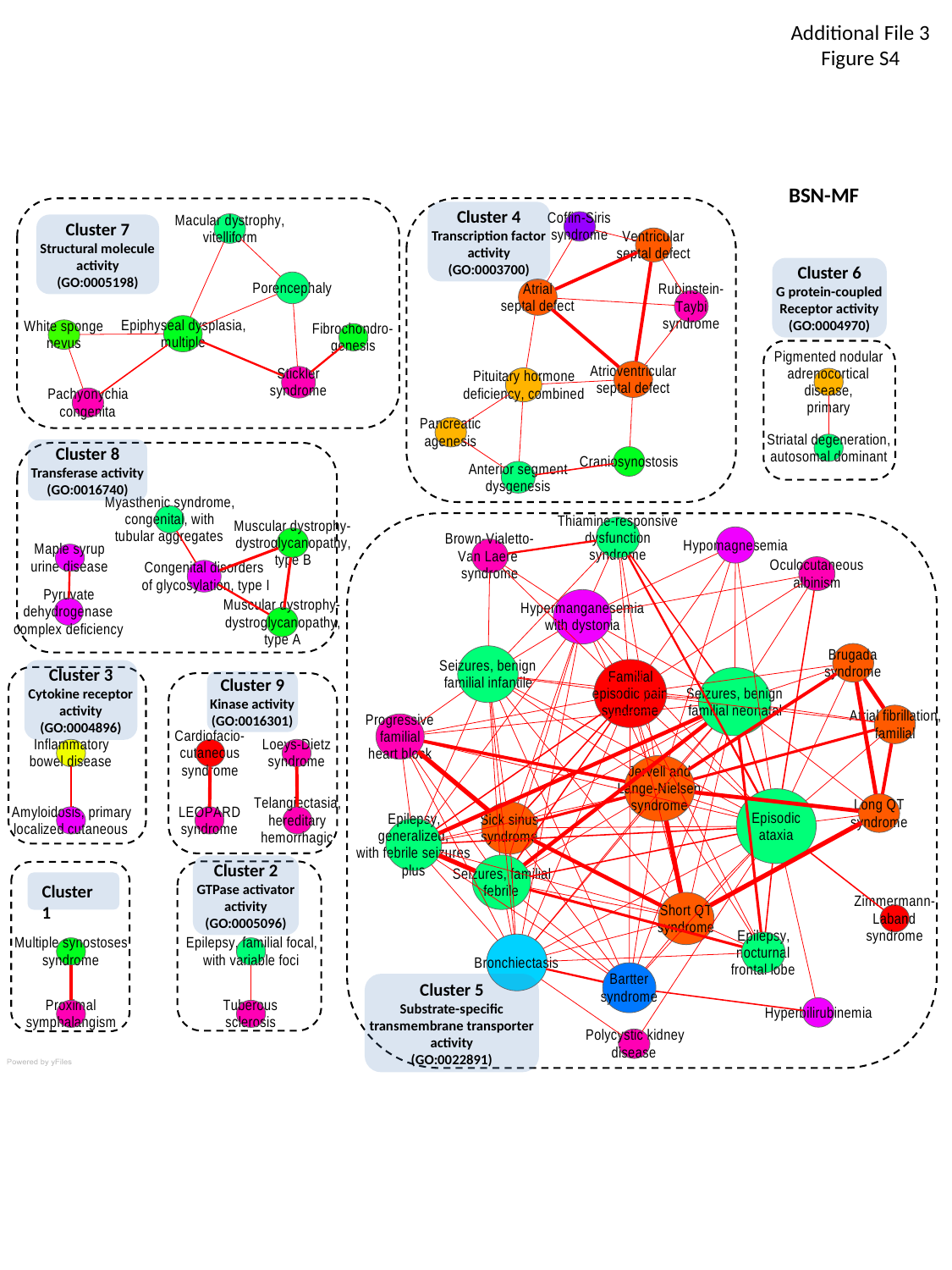

Additional File 3
Figure S4
BSN-MF
Cluster 4
Transcription factor
activity
(GO:0003700)
Cluster 7
Structural molecule
activity
(GO:0005198)
Cluster 6
G protein-coupled
Receptor activity
(GO:0004970)
Cluster 8
Transferase activity
(GO:0016740)
Cluster 3
Cytokine receptor
activity
(GO:0004896)
Cluster 9
Kinase activity
(GO:0016301)
Cluster 2
GTPase activator
activity
(GO:0005096)
Cluster 1
Cluster 5
Substrate-specific
transmembrane transporter
activity
(GO:0022891)
